# Somatostatin receptor 2 targeting in small cell lung carcinoma: perspectives

**DOI:** 10.18632/oncotarget.27107

**Published:** 2019-07-30

**Authors:** Jonathan M. Lehman, Pierre P. aMassion

**Affiliations:** ^1^ Division of Medical Oncology, Thoracic Program, Vanderbilt Ingram Cancer Center, Nashville, TN, USA; ^2^ Thoracic Program, Vanderbilt Ingram Cancer Center, Nashville, TN, USA; ^3^ Veterans Affairs, Tennessee Valley Healthcare System, Nashville Campus, Nashville, TN, USA

**Keywords:** small cell lung cancer, somatostatin receptor 2, antibody-drug conjugate, peptide drug conjugate, perspectives

## Abstract

Small Cell lung carcinoma (SCLC) is the most lethal and aggressive subtype of lung cancer. Novel targeting approaches and agents are desperately needed. In this perspectives, we briefly explore recent data published in the International Journal of Cancer suggesting Somatostatin Receptor 2 (SSTR2) as a viable target for SCLC, summarize the current clinical trial space, and describe promising new research and clinical directions for Somatostatin Receptor 2 targeting in SCLC.

## INTRODUCTION

Small cell lung carcinoma (SCLC) is the most lethal and aggressive subtype of lung cancer and taken by itself is the 6th leading cause of cancer death ahead of all lymphoma or leukemia. Effective novel therapeutics in SCLC have lagged behind other carcinomas which led to the designation of SCLC as a recalcitrant cancer [1]. Novel targeting agents that can overcome the cellular heterogeneity and chemoresistance associated with SCLC recurrence are sorely needed.

Somatostatin Receptor 2 is the best characterized G protein coupled receptor in its family and modulates GI motility and has multiple roles via adenylate cyclase, calcium influx and has effects on cell cycling angiogensisis, apopotosis and growth factor signaling. Its activation is used clinically to reduce tumor growth and prolong survival and quality of life in low grade neuroendocrine carcinomas. However, in *Somatostatin Receptor 2 signaling* promotes growth and survival in High Grade Neuroendocrine Lung Cancer [2], we showed that SSTR2 is highly expressed in both primary SCLC and cell lines and serves a vital pro survival role in high grade neuroendocrine carcinomas. We are further investigating the mechanism of this role, but it likely stems from both metabolic modulation in the highly active SCLC tumor and lack of apoptotic signaling machinery with loss of p53 and Rb in high grade neuroendocrine tumors. These results have significant potential clinical impact given that both agonist and antagonists of this pathway are being use for targeting and signal modulation in neuroendocrine tumors. Here, we highlight active or recent clinical trials using SSTR2 as a target and discuss the implications of this work for these studies.

### SSTR2 based targeting in small cell lung carcinoma: the current state of the art

There are two primary mechanisms of treatment efficacy in SSTR2 based studies for SCLC and other neuroendocrine models. One relies on SSTR2 signaling induced changes to induce apoptosis or cell cycle arrest mediated by activity at p53 and p27.

Given the lack of p53 and Rb, SSTR2 agonist based signaling approaches are unlikely to have therapeutic efficacy in SCLC. Indeed, multiple clinical trials over many years have attempted to use SSTR2 agonists to modulate signaling and have been unsuccessful [2, 3]. SSTR2 antagonists would be another potential option for exploration and we are engaged in this research, however, it is unclear whether the current SSTR2 “antagonists” are true signaling antagonists or may actually be partial agonists for this pathway. Indeed, Somatostatin “antagonists” have been shown to have partial agonist activity [4]. Accounting for the diversity in receptors with 5 potential receptors and the diverse signaling environment, predicting cellular responses in high grade tumors to SSTR2 agonism or antagonism is challenging. This is further complicated by the fact that there are a dearth of studies using SCLC models for SSTR2 signaling. Target development screens for SSTR2 routinely use signaling intact models such as HEK293 cells with Somatostatin over expression with intact p53 or Rb function which is not characteristic of SCLC [5, 6].

A second primary mechanism which has been clinically efficacious is the use of SSTR2 as a targeting molecule to deliver a cytotoxic or radioactive payload. This technique has been proven clinically effective in the NETTR-1 trial with low grade neuroendocrine tumors and is a topic of active exploration in current clinical trials (Table 1). Our work shows high levels of SSTR2 expression in many SCLC tumors, however, based on IHC, not every cell is SSTR2 positive and spotty staining is possible. The strong linkage identified in this work between SSTR2+ and NEUROD1+ cells also supports work to date suggesting diverse populations of cellular subtypes in different patients and potentially within the same tumor [7, 8]. This suggests potential heterogeneity in tumor response from tumor to tumor and also suggests that antibody drug conjugates targeted at SSTR2 may only lead to enriched killing of SSTR2+ cell subpopulations. Similar issues are seen with other antibody drug conjugate based approaches such as Dll3 targeting [9, 10]. One potential approach to address this issue is theragnostic based tumor imaging to assess SSTR2 positivity. However, the initial studies in this area were performed in low grade NET without the same vascular or inflammatory milieu of SCLC. False positive results have been seen with gallium-DOTATATE both for other carcinomas such as renal cell carcinoma, and in NSCLC or inflammatory or vascular lesions [11, 12]. Again, a scarcity of pre-clinical SCLC models in this area is concerning and a topic of active research. Recent studies on the heterogeneity of SCLC cell lines also raise concerns that SCLC cells lines may insufficiently reflect the heterogeneity of this disease. Patient derived xenografts or GEMM models may better reflect the diversity of these tumors in human patients. It is vitally important to incorporate lessons learned form prior SSTR2 clinical trials and testing into the study of novel neuroendocrine targeting agents in SCLC.

**Table 1:** SSTR2 targeted recent and active clinical trials for small cell lung cancer and high grade neuroendocrine carcinomas

Trial name	Target	Payload	Trial status	Identifier	Last update
Phase I/II Trial of Rhenium 188-P2045 in Small Cell Lung Cancer and other Advanced Neuroendocrine Carcinomas	SSTR2 (peptide agonist)	Rhenium-188	Withdrawn	NCT02030184	2017
PEN-221 in Somatostatin Receptor 2 Expressing Advanced Cancers including Neuroendocrine and Small Cell Lung Cancers	SSTR2 (peptide agonist)	DM-1	Recruiting	NCT02936323	2019
Evaluate the Safety, Tolerability, Biodistribution and Anti Tumour Activity of 177LU-OPS201 with Companion Imaging 68G-OPS202 PET/CT in Previously Treated Subjects with locally Advanced or Metastatic Cancers Expressing Somatostatin Receptor 2 (SSTR2)	SSTR2 (peptide antagonist)	177Lu	Recruiting	NCT03773133	2019
Nivolumab and 177Lu-DOTA0-Tyr3-Octreotate for Patients with Extensive Stage Small Cell Lung Cancer	SSTR2 (peptide agonist)	177Lu	Active, not recruiting	NCT03325816	2018
A Trial to Assess the Safety and Effectiveness of Lutetium-177 Octreotate Therapy in Neuroendocrine Tumours	SSTR2 (peptide agonist)	177Lu	Recruiting	NCT01876771	2017

### Future directions for SSTR2 and SCLC

The recent data presented in *Somatostatin Receptor 2 signaling* promotes growth and survival in High Grade Neuroendocrine Lung Cancer strongly supports targeting SSTR2 signaling and expression as a viable target for SCLC tumors. Multiple clinical have arisen targeting SSTR2+ SCLC cells. However, the field needs a better mechanistic understanding of how SSTR2 signaling varies between low and high-grade neuroendocrine tumors to best uncover novel agents. Furthermore, understanding the heterogeneity of SCLC tumors and how SSTR2+ subsets contribute and signal in the tumor is vital to predict which patients will have the best response to treatment. These are areas of active investigation; in the interim, new clinical trials in this space should use both imaging and immunohistochemistry (IHC) based approaches to select patients for treatment and incorporate best antibody drug conjugate (ADC) practices to minimize off target binding and maximize tumor death in both the target cells and neighboring SSTR2- SCLC cells. Another innovative approach is to combine immunotherapy with targeted agents in SCLC [13]; however, SSTR2 is expressed in inflammatory cells and T cells and the interplay between SSTR2 targeting and immune response is unclear [14, 15]. Expanding foundational research into these questions and using best practices from correlative research and ADC design will best capitalize on the need for SSTR2 survival signaling in SCLC tumors and may lead to improved therapeutic options for this devastating disease.
